# Total Synthesis of Natural Terpenoids Enabled by Cobalt Catalysis

**DOI:** 10.3389/fchem.2022.941184

**Published:** 2022-06-15

**Authors:** Shu Xiao, Likun Ai, Qichang Liu, Baihui Yang, Jian Huang, Wei Xue, Yang Chen

**Affiliations:** State Key Laboratory Breeding Base of Green Pesticide and Agricultural Bioengineering, Key Laboratory of Green Pesticide and Agricultural Bioengineering, Ministry of Education, Research and Development Center for Fine Chemicals, Guizhou University, Guiyang, China

**Keywords:** cobalt-mediated, total synthesis, natural terpenoids, asymmetric catalysis, research advanced

## Abstract

Transition metal catalysis plays an essential role in the total synthesis of natural products. Cobalt-mediated asymmetric catalysis has successfully been used as a primary or a secondary step in the total synthesis of natural products, especially terpenoids. Terpenoids represent one of the most prominent families among various categories of natural products, attracting immense attention due to their promising physiological activities. This review summarizes the recent advances toward the total synthesis of terpenoids by cobalt-mediated asymmetric catalysis, which may shed some light on their future synthetic efforts toward natural pesticides such as celanguline, azadirachtin, etc.

## Introduction

Cobalt is a transition metal widely distributed on the earth. It has been used for organic synthesis in catalytic reactions for almost a century. Roelen (1938) achieved the hydroformylation alkene under Co_2_(CO)_8_-catalyzed conditions, which was a seminal work highlighting the impact of organometallic cobalt catalysts on selective organic transformations. It is worth mentioning that terpenoids are integral parts of natural products, widely distributed in plants, microbes, marine life, and some insects. The semi-synthesis and total synthesis of terpenoids gradually became one of the essential hotspots in the 20th century due to their various chemical structures and significant biological activities. In the following decades, great efforts have been made to explore novel strategies to synthesize the critical intermediates of terpenoids by catalyzing cobalt. Various studies have shown that cobalt catalysts expand the scope and range of organic methods, particularly in synthesizing small rings and enantioselective reactions. In this context, recent reviews summarized the enantioselective cobalt-catalyzed transformations (Pellissier and Clavier, 2014), catalytic activation of olefins using cobalt complex (Shigehisa, 2018), and metal-hydride hydrogen atom transfer (MHAT) reactions in natural product synthesis (Wu and Ma, 2021). However, there is a lack of a systematic and comprehensive summary of the application of cobalt catalysis in the total synthesis of terpenoids. Given the continuous progress in this research field, this review summarizes the total synthesis of terpenoids by asymmetric cobalt catalysis over the past few decades. It introduces aspects of hydration, hydrovinylation, hydroperoxidation, isomerization, and cycloaddition by cobalt-catalyzed. Partial functional group manipulation steps in the synthetic routes are omitted due to space limitations.

## The Total Synthesis of Terpenoid Enabled by Cobalt-Catalyzed

### Hydration

Asymmetric hydration represents a powerful tool for converting alkenes into valuable and chiral building blocks for organic synthesis. It is a fundamental challenge for all hydration reactions to control the stereoselectivity of the alkene. We will systematically introduce applications of olefins hydration triggered through cobalt catalysis. One of the most widely employed cobalt radical reactions is the Mukaiyama hydration, a mild method to construct the C-O bonds across double bonds. Many reports show that cobalt-mediated Mukaiyama hydration of olefins has numerous applications due to its high regio- and chemoselectivity.

**FIGURE 1:**
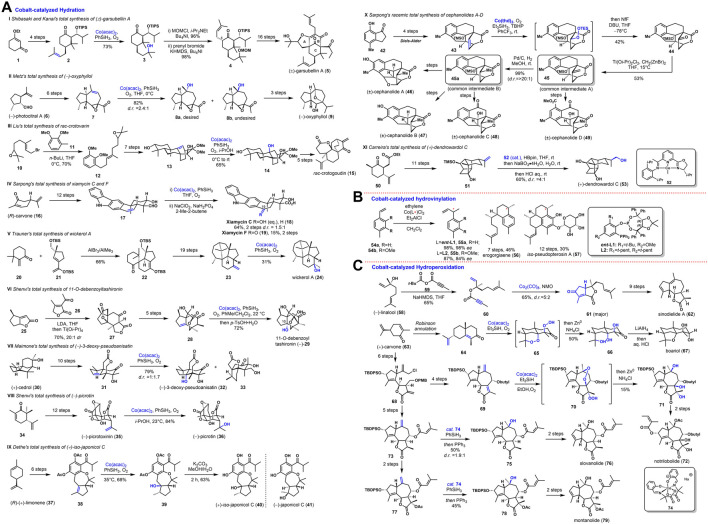
Cobalt-catalyzed Hydration, Hydrovinylation, and Hydroperoxidation.

Shibasaki and Kanai (Kuramochi et al., 2005) achieved the first total synthesis of garsubellin A (5) using a cobalt-catalyzed Mukaiyama hydration. As shown in Figure 1A-I, isopropenyl ketone 2 was provided from commercially available cyclohexanone 1 in four steps. To complete the construction of the A-B rings in the later stage, the authors firstly protected the prenyl group under the conditions (Co(acac)_2,_ PhSiH_3,_ and O_2_) to obtain tertiary alcohol 3, followed by treatment of 3 with MOMCl; a second prenyl group was introduced to C-4 at the axial position to give coupling compound 4. Later, (±)- garsubellin A (5) was prepared *via* a sixteen-step conversion from 4.

Metz (Zahel and Metz, 2013) finished the synthesis of (−)-oxyphyllol (9) by a regio- and diastereoselective Co (II)-catalyzed hydration of olefin. As shown in Figure 1A-II, the total syntheses started from (−)-photocitral A (6). By a six-step conversion, the intermediate 7 was prepared. Under Co-catalyzed Mukaiyama hydration, a diastereomeric mixture of alcohol 8a and 8b was furnished with an 82% overall yield. (−)-Oxyphyllol (9) was then synthesized from 8a in three steps.

Liu (Song et al., 2015) applied Mukaiyama hydration in the total synthesis of atisane-type diterpenoids. As shown in Figure 1A-III, their synthesis started with 12, which could be accessed by coupling epoxy geranyl bromide 10 and 1,2-dimethoxy-3-methylbenzene 11. Afterward, a seven-step conversion furnished tetracyclic 13, followed by Mukaiyama hydration reaction with Co(acac)_2_ as the catalyst to afford triol 14 in 65% yield. After five steps, the total synthesis of *rac*-crotovarin (15) was accomplished.

Sarpong (Pfaffenbach et al., 2019) synthesized indole sesquiterpenoids xiamycins A, C, F, H and oridamycin A. As shown in Figure 1A-IV, aldehyde 17 was prepared from (*R*)-carvone (16) after twelve steps as a common late-stage intermediate applied to synthesize several xiamycin congeners. Mukaiyama hydration of 17 with Co(acac)_2_ as the catalyst followed by Pinnick oxidation obtained a pair of diastereomers xiamycin C (18) and 19-*epi*-xiamycin C (*d.r.* = 1.5:1) in 64% yield over two steps, and xiamycin F (19) in 15% yield.

Trauner (Liu and Trauner, 2017) reported the synthesis of the antiviral diterpene, wickerol A (24). As shown in Figure 1A-V, with enone 20 and diene 21 as start material, tricylic enol silyl ether 22 was constructed *via* Diels–Alder cycloaddition. Whereafter, tetracyclic compond 23 was obtained from 22 in nineteen steps. The authors finished the total synthesis of wickerol A (24) at the last step of the Mukaiyama hydration of olefin *via* CpCo(CO)_2_ catalyst with a 31% yield.

Shenvi (Ohtawa et al., 2017) prepared neurotrophic sesquiterpenes, 11-*O*-debenzoyltashironin (29). As shown in Figure 1A-VI, The synthesis commenced with the construction of tetracycle 27, which was accessed through a cycloaddition between cyclopentanobutenolide 25 and butenolide 26. The seven-membered lactone 28 could be constructed in five steps containing a Dieckmann-type condensation from 27. The authors suspected that the stereochemistry of hydration products might be affected by steric shielding by the C10 alcohol. Thus, the authors applied Mukaiyama hydration *via* Co(acac)_2_ catalyst in the last stage toward the total synthesis of 11-*O*-debenzoyltashironin (29), followed by hemiacetalization with *p-*TsOH·H_2_O to transform the *trans*-hydrindane skeleton with tertiary alcocyclic in 72% yield.

Maimone (Hung et al., 2019) explored a terpene feed stock-based oxidative synthetic approach to synthesize the *Illicium* sesquiterpenes. As shown in Figure 1A-VII, the alkene 31 was prepared from (+)-cedrol (30) in ten steps. Under the presence of Co(acac)_2_, PhSiH_3,_ and O_2_, 31 could be converted to 3-deoxy-pseudoanisatin (32) and its epimer 33 in a 1:1.7 ratio with a 50% yield *via* a radical hydration reaction.

As shown in Figure 1A-VIII, Shenvi (Crossley et al., 2020) accomplished the total synthesis of (−)-picrotoxinin (35) from dimethyl-(*R*)-carvone 34 in 12 steps. Under Mukaiyama hydration conditions, Co(acac)_2_ and PhSiH_3_ in *i*-PrOH under O_2_, (−)-picrotin (36) was isolated in one step and 84% yield.

Dethe (Dethe and Nirpal, 2021) described the enantiospecific total synthesis of japonicol C (41). The allyl alcohol 38 could be advanced to (*R*)-(+)-limonene (37) in six steps. As shown in Figure 1A-IX, treatment of 38 with Co(acac)_2_, PhSiH_3,_ O_2,_ and THF, generated the stereospecific product 39 in 68% yield. Although it was an unsuccessful synthesis of japonicol C (41), it provided (+)-*iso*-japonicol C (40) after deacetylation of 39 in 63% yield. The authors speculated that the stereochemical outcome of the Mukaiyama reaction could be due to the Co-H hydride approaching from a less hindered side. To overcome the obstacle, they took advantage of Pd(OH)_2_/C-catalyzed isomerization/hydrogenation to furnish (−)-japonicol C (41) from allyl alcohol 38.

As shown in Figure 1A-X, a divergent total synthesis of cephalotane-type nor-diterpenoids cephanolides A-D (46–49) was reported by Sarpong (Haider et al., 2021). Their synthesis commenced from the commercially available 7-hydroxy-4-methylindanone 42, which could be converted to bridge lactone 43 *via* a four-step sequence with intramolecular Diels–Alder cycloaddition as a critical step. The common intermediates 45 and 45a containing the A/B/C/D/E rings of cephanolides A-D were synthesized by Mukaiyama hydration under Co(thd)_2_, O_2_, Et_3_SiH, and TBHP from 43. In addition, it is necessary to form 44 to use NfF and excess DBU, following converting ketone to an olefine 45 with Ti(O*i*-Pr)_2_Cl_2_ and Nystedt reagents, followed by Pd catalytic hydrogenation reduction to get common intermediate B (45a). In a word, the cephanolide A (46) was successfully constructed using 42 as the starting material and 45 as common intermediate in overall 14 steps; cephanolide B (47, 10 steps), cephanolide C (48, 8 steps), and cephanolide D (49, 14 steps) were accessed through late-stage oxygenation from a commercially available indanone (42) and 45 as a comm intermediate A.

Carreira (Wolleb and Carreira, 2017) reported the asymmetric total synthesis of (+)-dendrowardol C (53). As shown in Figure 1A-XI, the fused tetracyclic carbon skeleton of 51 was prepared from known ester 50 in eleven steps. They initially tried to construct the primary alcohol 53 through hydroboration-oxidation reaction with the desired configuration. However, they always gave the 1:1 mixture of diastereomers. Towards this end, treatment of 51 with chiral Co^I^ (52) and HBpin, followed by oxidative and global deprotection, afforded the natural product (+)-dendrowardol C (53) in a diastereomeric ratio of 4:1 with 60% yield.

### Hydrovinylation

Cobalt-catalyzed asymmetric hydrovinylation has the potential to control the *R/S* stereocentres of C–C bond formations. Moreover, the choice of the Co-catalyst enables diverse products. The method has been applied in the total synthesis of terpenoids. Co reacted as radical metal hydrides or hydrogen atom transfer (HAT) reagents, which prefer to add hydrogen to the less sterically hindered olefin position to form the most stable radical.

Schmalz (Movahhed et al., 2021) developed a new method of enantioselective cobalt-catalyzed hydrovinylation to introduce chiral olefins. As shown in Figures 1B, taking advantage of the novel method as the chirogenic step the asymmetric hydrovinylation of vinyl-arenes 54a/54b was performed utilizing Co(L*)Cl_2_ as a catalyst under the atmosphere of ethylene and Et_2_AlCl, providing vinyl-arenes 55a/55b with remarkable efficiency and excellent enantioselectivity. The total synthesis of (+)-erogorgiaene (56) was achieved in only seven steps with a 46% overall yield from 4-methyl-styrene 54a. Moreover, the *iso*-pseudopterosin A (57) was prepared in 12 steps with a 30% overall yield from 54b, which proved to be equally anti-inflammatory as a mixture of natural pseudopterosins.

### Hydroperoxidation

As shown in Figure 1C, Maimone (Hu et al., 2019) reported the synthesis of complex guaianolide sesquiterpenes. For sinodielide A (62), treatment of (−)-linalool (58) with NaHMDS and 59 was converted into ester 60, followed by underwent smooth Pauson-Khand reaction using dicobalt octacarbonyl (Co_2_(CO)_8_) gave bicyclic lactone 61. An additional nine steps gave 62, Wieland-Miescher Ketone analogue 64 was obtained by Robinson annulation starting with (+)-carvone (63). Then 64 was converted to triol 66 by a tandem cobalt-catalyzed hydroperoxidation *via* bis-peroxide intermediate 65, followed by Zinc powder reduction in a respectable 50% isolated yield. At the last step, boariol (67) was prepared using a stereoselective reduction with LiAlH_4_ and etherification. With the same procedure and conversion, the total synthesis of notrilobolide (72) was achieved in the overall 14 steps from (+)-carvone (63) as starting material *via* trien 69 and triol 71 as critical intermediates. Notably, the total syntheses of slovanolide (76) and montanolide (79) *via* cobalt-catalyzed Mukaiyama-type hydration individually from common intermediate 73.

### Isomerization of Olefins

**FIGURE 2:**
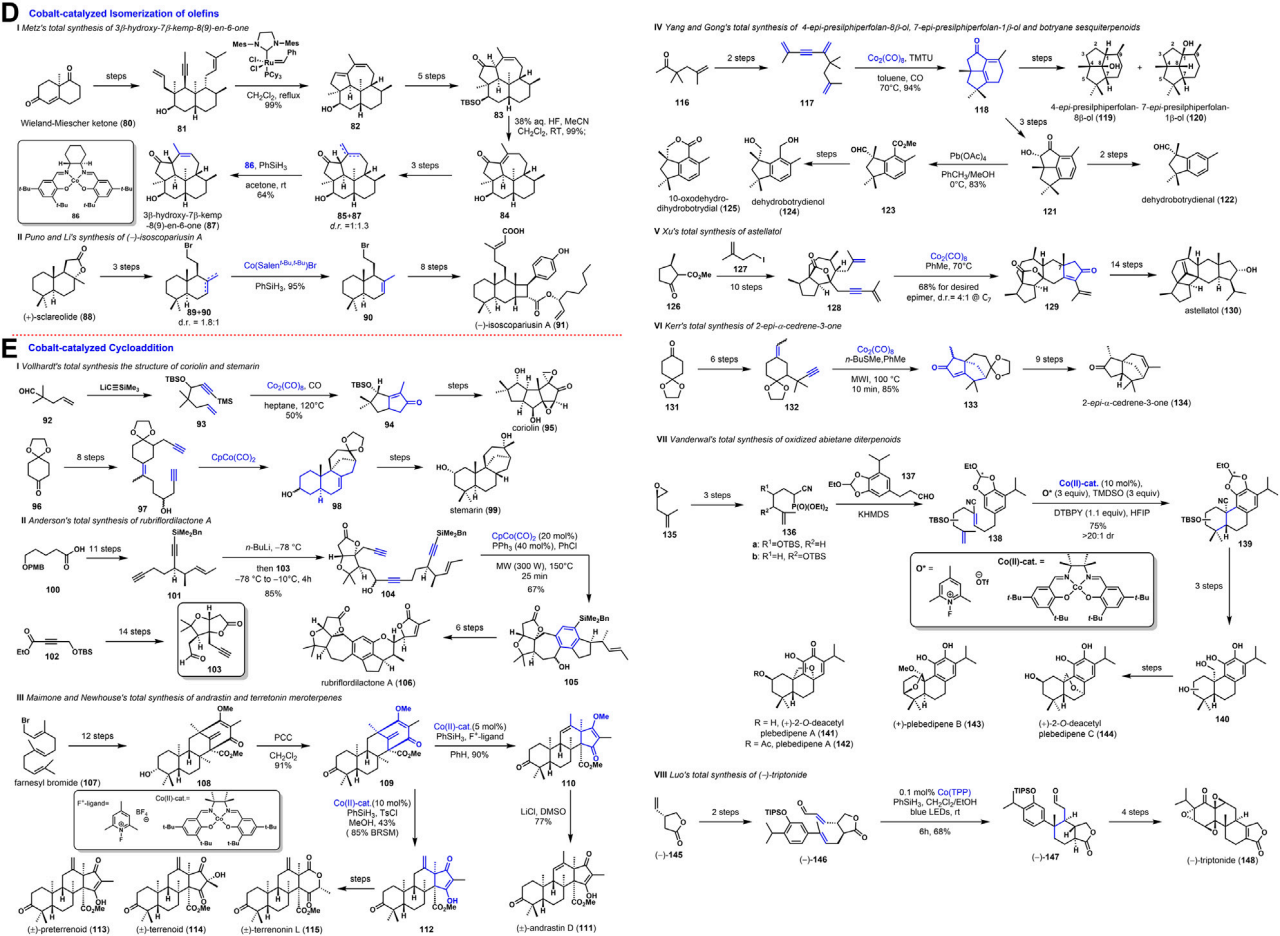
Cobalt-catalyzed Isomerization of olefins, and Cycloaddition.

The isomerization of double bonds has attracted attention during the last decade due to the growing importance of selectively shifting this synthetically essential functionality within a molecule. Cobalt complexes have also been utilized for the migration transposition of double bonds along a carbon chain. Hilt (Puenner et al., 2012) realized a transposition of a terminal alkene towards an internal alkene by cobalt catalyzed, and it was applied in a particular total synthesis of terpenoids later. As shown in Figure 2D-I, Metz (Wang et al., 2017) accomplished the first total synthesis of 3β-hydroxy-7β-kemp-8 (9)-en-6-one (87), which was isolated from the soldier defense secretion of the higher termites Nasutitermes octopolis. The Wieland-Miescher ketone 80 could be advanced to β-oriented alcohol 81 in multi-step transformation, giving rise to the requisite tetracyclic dienol 82 *via* Ru catalyzed domino metathesis. Following five steps of 82 generated the β, γ-unsaturated ketone 83. Later, desilylation and olefin isomerization with HF led to conjugated enone 84, which could be converted into the natural product in late continuous three steps. However, the byproduct of exocyclic olefin isomer 85 made the synthetic route more inefficient. Thus, they explored a cobalt-catalyzed isomerization strategy to install the β, γ-unsaturated ketone moiety from this tetracyclic compound 85 by hydrogen atom transfer with 86 as catalysis, provided the 3β-hydroxy-7β-kemp-8 (9)-en-6-one (87) in good yield.

Last year, Puno and Li (Yan et al., 2021) synthesized immunosuppressive meroditerpenoid, (−)-isoscopariusin A (91). As shown in Figure 2D-II, the synthesis commenced with the construction of mixed olefins 89 and 90 (*d.r.* = 1.8:1), which was accessed from (+)-sclareolide (88) *via* reduction, elimination and bromination. Inspired by Shenvi’s protocol (Crossley et al., 2014) for olefin isomerization through Co(Salen^*t-*Bu,*t-*Bu^)Br catalyzed, the exocyclic olefin 89 was converted to cycloolefin 90. Eventually, a following eight-step conversion achieved the total synthesis of (−)-isoscopariusin A (91) on a gram scale.

### Cycloaddition

Cobalt catalyzed cycloaddition reaction has been used to construct polycyclic skeleton in total synthesis of terpenoids showed some case studies, such as [2+2+1] and [2+2+2] cycloaddition. The Pauson–Khand reaction is a metal-mediated [2+2+1] cycloaddition of an alkene, an alkyne, with carbon monoxide to construct an α, β-cyclopentenone skeleton.

Magnus (Exon and Magnus, 1983) employed intramolecular alkene-alkyne dicobaltocta-carbonyl mediated cyclopentenone cyclization to synthesize the antitumor sesquiterpene coriolin (95). The dicobalt octacarbonyl strategy for the stereoselective synthesis of hydroxylated bicyclo [3.3.0] enones provides a direct method of making many other natural and unnatural cyclopentanoid products. The unique ability of the [2+2+2] cycloaddition to form several new bonds within one step and thereby assemble smaller synthons to a bigger core structure predestinates this reaction to be applied to synthesize natural products. As shown in Figure 2E-I, Vollhardt (Germanas et al., 1991) employed a novel application of the cobalt-catalyzed cycloaddition to synthesize corioline (95) and stemarin (99). In the total synthesis of corioline (95), the cobalt-catalyzed Pauson–Khand reaction was used to construct the key intermediate 94. Moreover, cobalt mediated [2+2+2] cycloaddition reaction with CpCo(CO)_2_ played an essential role in the total synthesis of stemarin (99), in which intermediates 98 were constructed with high efficiency and stereoselectivity.

Rubriflordilactones A and B from *Schisandra rubriflora* have attracted increasing attention because of their intriguing structures and promising anti-HIV activity. As shown in Figure 2E-II, Anderson (Goh et al., 2015) (Chaubet et al., 2017) completed the total synthesis of rubriflordilactone A (100) *via* a cobalt-catalyzed [2+2+2] cycloaddition as a pivotal step to close the key aromatic C-ring. This total synthesis takes 5-OPMB-pentanoic acid 100 as the starting material; Diyne 101 was prepared after a eleven-step sequence. Meanwhile, the cyclized precursor 104 was assembled with great facility *via* fragment coupling by 101 and bicyclic lactone alkyne 103, which was prepared from commercially available propargyl silicon 102 *via* fourteen steps. With an efficient cobalt-catalyzed [2+2+2] cycloaddition, the aromatic C-ring of intermediate 105 with pentacyclic core skeleton was constructed and followed a six-step transformation toward the total synthesis of rubriflordilactone A (106).

As shown in Figure 2E-III, in the total synthesis of andrastin and terretonin meroterpenes in 2017, Maimone and Newhouse (Xu et al., 2017) employed a strategy of purely radical-based homoallyl-type rearrangement/HAT to forge protoaustinoid bicyclo [3.3.1] nonane nucleus. Cycloaddition substrate 109 could be conveniently prepared by PCC oxidation from tetracyclic block 108, which was obtained from commercially available farnesyl bromide 107 in twelve steps. As a common intermediate, 109 was converted to hydroxymethyl ether cyclopentenone 110 and iso-cyclopentenone 112 by cobalt-catalyzed intramolecular cycloaddition reaction under the action of 5% mol catalyst and 10% mol catalyst, respectively. After cyclopentenone isomerization, the total synthesis of (±)-andrastin D (111) was accomplished. On the other hand, (±)-preterrenoid (113), (±)-terrenoid (114), and (±)-terretonin L (115) were prepared by severe simple conversion processes.

As shown in Figure 2E-IV, Yang and Gong (Zhang et al., 2017) explored an approach of tandem Pauson–Khand and 6π-electrocyclization toward the total syntheses of 4-*epi*-presilphiperfolan-8β-ol (119) and 7-*epi*-presilphiperfolan-1β-ol (120). The preparation of cyclization precursor (117) was achieved from commercially available ketone (116) in a two-step. Under the condition of a catalytic amount of Co_2_(CO)_8_ and TMTU by the Pauson–Khand reaction, tricycle ketone 118 was obtained in excellent yield (94%). As a common intermediate, ketone 118 was transformed to 4-*epi*-presilphiperfolan-8β-ol (119) and 7-*epi*-presilphiperfolan-1β-ol (120) in a few steps reaction. Next year, Yang and Gong (Zhang et al., 2019) continued their previous work by the Co-TMTU-catalyzed tandem Pauson-Khand and 6π-electrocyclizationreactions, the total syntheses of three botryane sesquiterpenoids: dehydrobotrydienal (122), dehydrobotrydienol (124) and 10-oxodehydrodihydrobotrydial (125) were delivered from the common intermediate 118.

As shown in Figure 2E-V, in the synthetic studies towards astellatol (130), Xu (Zhao et al., 2018) used a cobalt-catalyzed Pauson-Khand cycloaddition to install the right-hand side scaffold of the sesterterpenoid. The cycloaddition precursor 128 could be advanced from chiral synthon 126 and the homoallylic iodide 127 as start materials in teen steps. Following [2+2+1], cycloaddition was carried out successfully with Co_2_(CO)_8_ in the toluene under the heating condition to give cyclopentenone 129. Finally, the authors accomplished the first and enantiospecific total synthesis of the rare sesterterpenoid, astellatol (130) in 25 steps (0.63% overall yield) from 126.

As shown in Figure 2E-VI, Kerr et al. (2018) accomplished the total synthesis of 2-*epi*-α-cedrene-3-one (134) *via* a cobalt-catalyzed Pauson-Khand reaction, which was isolated from the essential oil of *Juniperus thurifera*. Because the reaction required higher activation energy, the cycloaddition precursor 132 transformed to cyclopentenone 133 *via* a microwave-assisted, cobalt catalytic, Pauson-Khand reaction, which is used to construct the intriguing tricyclic core of the target molecule 134 following nine steps conversion.

As shown in Figure 2E-VII, Vanderwal (Vrubliauskas et al., 2021) systematically studied that Co-catalyzed MHAT-initiated (metal-catalyzed hydrogen atom transfer, MHAT) radical bicyclization was uniquely effective in synthesizing polycyclic terpenoids from polyene. The authors researched the utility of these reactions in synthesizing three aromatic abietane diterpenoids. A known epoxide 135 as the starting material was transformed to phosphonate 136 with different cyanophosphonate reagents in three steps. Then the cyclization precursor 138 was prepared by HWE olefination with aldehyde 137. By a Co(II)-catalyzed tandem polyene cyclization, delivered 139 with excellent stereochemical control in 75% yield. The common intermediate 140 was prepared by continuous reduction and deprotection in three steps, following severe transformations prepared (+)-2-*O*-deacetyl plebedipene A (141), (±)-plebedipene B (143), (+)-2-*O*-deacetyl plebedipene C (144) and plebedipene A (142).

As shown in Figure 2E-VIII, Luo (Fang et al., 2022) described the total synthesis of (−)-triptonide (148) based on a Co(TPP)-catalyzed hydrogen atom transfer (MHAT)-initiated radical cyclization. Starting from (*R*)-(−)-Taniguchi lactone (145), fragment coupling was achieved through two-step reaction to obtain conjugated alkenal (146). By Cobalt-catalyzed in the presence of the photoredox catalyst and visible light, 146 was carried out to afford cycloadduct 147 smoothly. After the subsequent four steps transformation, (−)-triptonide (148) was produced.

## Conclusion and Outlook

Cobalt-mediated asymmetric catalysis is a powerful method to functionalize olefins. The application of cobalt catalysts in [2+2+2] cycloaddition reactions of alkynes, alkenes, and nitriles to afford substituted benzenes, cyclohexadienes, and an extensive array of derivatives has been an active field of research over the last centuries. During the last decade, the heavier group congeners also significantly impacted this research field; especially cobalt catalysis is often the first choice when planning to include a cyclotrimerisation reaction in a synthetic sequence. Many reports have shown that cobalt-catalyzed could form C-C or C-X bonds. However, many of the already explored synthetic methods still represent an unsolved challenge for isolated natural products, as they remain inaccessible by the reported strategies. Such studies will benefit the detailed investigations of the mechanism of cobalt catalyzed and the application potential of natural products. The review may shed some light on future synthetic efforts on the cobalt-mediated total synthesis of terpenoids toward natural pesticides, such as celanguline, and azadirachtin.

## References

[B1] Chaubet G. Goh S. S. Mohammad M. Gockel B. Cordonnier M.-C. A. Baars H. (2017). Total Synthesis of the Schisandraceae Nortriterpenoid Rubriflordilactone A. Chem. Eur. J. 23, 14080–14089. 10.1002/chem.201703229 28768051 PMC5656881

[B2] Crossley S. W. M. Barabé F. Shenvi R. A. (2014). Simple, Chemoselective, Catalytic Olefin Isomerization. J. Am. Chem. Soc. 136, 16788–16791. 10.1021/ja5105602 25398144 PMC4277770

[B3] Crossley S. W. M. Tong G. Lambrecht M. J. Burdge H. E. Shenvi R. A. (2020). Synthesis of (−)-Picrotoxinin by Late-Stage Strong Bond Activation. J. Am. Chem. Soc. 142, 11376–11381. 10.1021/jacs.0c05042 32573211 PMC8011636

[B4] Dethe D. H. Nirpal A. K. (2021). Enantiospecific Total Synthesis of (−)-Japonicol C. Org. Lett. 23, 2648–2653. 10.1021/acs.orglett.1c00560 33728920

[B5] Exon C. Magnus P. (1983). Stereoselectivity of Intramolecular Dicobalt Octacarbonyl Alkene-Alkyne Cyclizations: Short Synthesis of Dl-Coriolin. J. Am. Chem. Soc. 105, 2477–2478. 10.1021/ja00346a063

[B6] Fang X. Zhang N. Chen S. C. Luo T. Luo T. Chen S. C. (2022). Scalable Total Synthesis of (−)-Triptonide: Serendipitous Discovery of a Visible-Light-Promoted Olefin Coupling Initiated by Metal-Catalyzed Hydrogen Atom Transfer (MHAT). J. Am. Chem. Soc. 144, 2292–2300. 10.1021/jacs.1c12525 35089705

[B7] Germanas J. Aubert C. Vollhardt K. P. C. (1991). One-step Construction of the Stemodane Framework via the Cobalt-Catalyzed Cyclization of Monocyclic Enynes: a Formal Total Synthesis of Stemodin. J. Am. Chem. Soc. 113, 4006–4008. 10.1021/ja00010a061

[B8] Goh S. S. Chaubet G. Gockel B. Cordonnier M.-C. A. Baars H. Phillips A. W. (2015). Total Synthesis of (+)-Rubriflordilactone A. Angew. Chem. Int. Ed. 54, 12618–12621. 10.1002/anie.201506366 PMC4643188 26337920

[B9] Haider M. Sennari G. Eggert A. Sarpong R. (2021). Total Synthesis of the Cephalotaxus Norditerpenoids (±)-Cephanolides A-D. J. Am. Chem. Soc. 143, 2710–2715. 10.1021/jacs.1c00293 33577317

[B10] Hu X. Musacchio A. J. Shen X. Tao Y. Maimone T. J. (2019). Allylative Approaches to the Synthesis of Complex Guaianolide Sesquiterpenes from Apiaceae and Asteraceae. J. Am. Chem. Soc. 141, 14904–14915. 10.1021/jacs.9b08001 31448610 PMC6818654

[B11] Hung K. Condakes M. L. Novaes L. F. T. Harwood S. J. Morikawa T. Yang Z. (2019). Development of a Terpene Feedstock-Based Oxidative Synthetic Approach to the Illicium Sesquiterpenes. J. Am. Chem. Soc. 141, 3083–3099. 10.1021/jacs.8b12247 30698435 PMC6563921

[B12] Kerr W. J. McLaughlin M. Paterson L. C. Pearson C. M. (2018). Total Synthesis 2-Epi-α-Cedren-3-One via a Cobalt-Catalysed Pauson-Khand Reaction. Tetrahedron 74, 5062–5068. 10.1016/j.tet.2018.06.032

[B13] Kuramochi A. Usuda H. Yamatsugu K. Kanai M. Shibasaki M. (2005). Total Synthesis of (±)-Garsubellin A. J. Am. Chem. Soc. 127, 14200–14201. 10.1021/ja055301t 16218611

[B14] Liu S.-A. Trauner D. (2017). Asymmetric Synthesis of the Antiviral Diterpene Wickerol A. J. Am. Chem. Soc. 139, 9491–9494. 10.1021/jacs.7b05046 28625047

[B15] Movahhed S. Westphal J. Kempa A. Schumacher C. E. Sperlich J. Neudörfl J. M. (2021). Total Synthesis of (+)‐Erogorgiaene and the Pseudopterosin A−F Aglycone via Enantioselective Cobalt‐Catalyzed Hydrovinylation. Chem. Eur. J. 27, 11574–11579. 10.1002/chem.202101863 34096655 PMC8456859

[B16] Ohtawa M. Krambis M. J. Cerne R. Schkeryantz J. M. Witkin J. M. Shenvi R. A. (2017). Synthesis of (−)-11-O-Debenzoyltashironin: Neurotrophic Sesquiterpenes Cause Hyperexcitation. J. Am. Chem. Soc. 139, 9637–9644. 10.1021/jacs.7b04206 28644021

[B17] Pellissier H. Clavier H. (2014). Enantioselective Cobalt-Catalyzed Transformations. Chem. Rev. 114, 2775–2823. 10.1021/cr4004055 24428605

[B18] Pfaffenbach M. Bakanas I. O'Connor N. R. Herrick J. L. Sarpong R. (2019). Total Syntheses of Xiamycins A, C, F, H and Oridamycin A and Preliminary Evaluation of Their Anti‐Fungal Properties. Angew. Chem. Int. Ed. 58, 15304–15308. 10.1002/anie.201908399 PMC6791741 31419367

[B19] Pünner F. Schmidt A. Hilt G. (2012). Up the Hill: Selective Double-Bond Isomerization of Terminal 1,3-dienes towards Z-1,3-Dienes or 2Z,4E-Dienes. Angew. Chem. Int. Ed. 51, 1270–1273. 10.1002/anie.201107512 22173992

[B20] Roelen O. (1944). ( to Chemische Verwertungsgesellschaft Oberhausen m.b.H.) German Patent DE 849548, 1938/1952; U.S. Patent 2327066, 1943. Chem. Abstr. 38, 3631.

[B21] Shigehisa H. (2018). Studies on Catalytic Activation of Olefins Using Cobalt Complex. Chem. Pharm. Bull. 66, 339–346. 10.1248/cpb.c17-01006 29607898

[B22] Song L. Zhu G. Liu Y. Liu B. Qin S. (2015). Total Synthesis of Atisane-type Diterpenoids: Application of Diels-Alder Cycloadditions of Podocarpane-type Unmasked Ortho-Benzoquinones. J. Am. Chem. Soc. 137, 13706–13714. 10.1021/jacs.5b08958 26434364

[B23] Vrubliauskas D. Gross B. M. Vanderwal C. D. (2021). Stereocontrolled Radical Bicyclizations of Oxygenated Precursors Enable Short Syntheses of Oxidized Abietane Diterpenoids. J. Am. Chem. Soc. 143, 2944–2952. 10.1021/jacs.0c13300 33555176 PMC8112877

[B24] Wang Y. Jäger A. Gruner M. Lübken T. Metz P. (2017). Enantioselective Total Synthesis of 3β-Hydroxy-7β-Kemp-8(9)-En-6-One, a Diterpene Isolated from Higher Termites. Angew. Chem. Int. Ed. 56, 15861–15865. 10.1002/anie.201708561 28960721

[B25] Wolleb H. Carreira E. M. (2017). Total Synthesis of (+)-Dendrowardol C. Angew. Chem. Int. Ed. 56, 10890–10893. 10.1002/anie.201705809 28691326

[B26] Wu J. Ma Z. (2021). Metal-hydride Hydrogen Atom Transfer (MHAT) Reactions in Natural Product Synthesis. Org. Chem. Front. 8, 7050–7076. 10.1039/d1qo01139a

[B27] Xu G. Elkin M. Tantillo D. J. Newhouse T. R. Maimone T. J. (2017). Traversing Biosynthetic Carbocation Landscapes in the Total Synthesis of Andrastin and Terretonin Meroterpenes. Angew. Chem. Int. Ed. 56, 12498–12502. 10.1002/anie.201705654 PMC5697905 28799296

[B28] Yan B. C. Zhou M. Li J. Li X. N. He S. J. Zuo J. P. (2021). (−)‐Isoscopariusin A, a Naturally Occurring Immunosuppressive Meroditerpenoid: Structure Elucidation and Scalable Chemical Synthesis. Angew. Chem. Int. Ed. 60, 12859–12867. 10.1002/anie.202100288 33620745

[B29] Zahel M. Metz P. (2013). A Concise Enantioselective Synthesis of the Guaiane Sesquiterpene (−)-oxyphyllol. Beilstein J. Org. Chem. 9, 20282025–20282032. 10.3762/bjoc.9.239 PMC3817518 24204414

[B30] Zhang Z. Li Y. Zhao D. He Y. Gong J. Yang Z. (2017). A Concise Synthesis of Presilphiperfolane Core through a Tandem TMTU-Co-Catalyzed Pauson-Khand Reaction and a 6π Electrocyclization Reaction (TMTU=Tetramethyl Thiourea). Chem. Eur. J. 23, 1258–1262. 10.1002/chem.201605438 28052485

[B31] Zhang Z. Zhao D. He Y. Yang Z. Gong J. (2019). Total Syntheses of Dehydrobotrydienal, Dehydrobotrydienol and 10-oxodehydrodihydrobotrydial. Chin. Chem. Lett. 30, 1503–1505. 10.1016/j.cclet.2019.03.033

[B32] Zhao N. Yin S. Xie S. Yan H. Ren P. Chen G. (2018). Total Synthesis of Astellatol. Angew. Chem. Int. Ed. 57, 3386–3390. 10.1002/anie.201800167 29364591

